# Prospects for the Use of Metal-Based Nanoparticles as Adjuvants for Local Cancer Immunotherapy

**DOI:** 10.3390/pharmaceutics15051346

**Published:** 2023-04-27

**Authors:** Irina Naletova, Barbara Tomasello, Francesco Attanasio, Victor V. Pleshkan

**Affiliations:** 1 Institute of Crystallography, National Council of Research, CNR, S.S. Catania, Via P. Gaifami 18, 95126 Catania, Italy; 2 Department of Drug and Health Sciences, University of Catania, V.le Andrea Doria 6, 95125 Catania, Italy; 3 Gene Immunooncotherapy Group, Shemyakin-Ovchinnikov Institute of Bioorganic Chemistry RAS, 117997 Moscow, Russia

**Keywords:** adjuvant, cancer immunotherapy, metal-based nanoparticles, local administration

## Abstract

Immunotherapy is among the most effective approaches for treating cancer. One of the key aspects for successful immunotherapy is to achieve a strong and stable antitumor immune response. Modern immune checkpoint therapy demonstrates that cancer can be defeated. However, it also points out the weaknesses of immunotherapy, as not all tumors respond to therapy and the co-administration of different immunomodulators may be severely limited due to their systemic toxicity. Nevertheless, there is an established way through which to increase the immunogenicity of immunotherapy—by the use of adjuvants. These enhance the immune response without inducing such severe adverse effects. One of the most well-known and studied adjuvant strategies to improve immunotherapy efficacy is the use of metal-based compounds, in more modern implementation—metal-based nanoparticles (MNPs), which are exogenous agents that act as danger signals. Adding innate immune activation to the main action of an immunomodulator makes it capable of eliciting a robust anti-cancer immune response. The use of an adjuvant has the peculiarity of a local administration of the drug, which positively affects its safety. In this review, we will consider the use of MNPs as low-toxicity adjuvants for cancer immunotherapy, which could provide an abscopal effect when administered locally.

## 1. Introduction

To achieve sustainable success in antitumor therapy, an effective and long-term effect that will wipe out all neoplasms in the body is required. Low-molecular-weight (LMW) compounds are not able to provide such an effect since their toxicity can also kill the organism, not just the tumor. The only approach that could most likely provide such an effect is the use of the immune system resources, which initially stand guard against tumors. If the immune system is working properly, it is able to protect a person from the misregulation of the molecular mechanisms involved in the functioning of a living cell that is part of a multicellular organism [1]. The prevailing model of cancer etiology is that errors in DNA replication lead to mutations in gene sequences, which are then copied in transcription and translation processes. At least some of these mutated proteins will be presented on the surface of the cancer cell via MHC class I molecules, leading to the cancer cell being identified by immune cells as abnormal and allowing it to be eliminated (refer, for example, to [2,3]). Impairment of such recognition for any reason can lead to the emergence and proliferation of a tumor.

Cancer immunotherapy is a way through which to restore the ability of the immune system to seek and destroy abnormal cancer cells by stimulating it with certain immunomodulatory approaches [4,5,6,7]. Here, we consider molecules that act on the pathways that regulate the immune system’s activity as immunomodulators. The immunotherapeutic treatment of different types of cancer with a growing number of immune checkpoint inhibitors (ICIs) has greatly improved the clinical management of advanced diseases (see the most recent studies [8,9,10,11]). However, only certain patients clinically respond to and benefit from the mentioned therapy. It would seem that in order to improve the proportion of responders, all that is required is to provide stronger immune stimulation. However, when it comes to the immune system, it is always worth remembering the risks of theralizumab, also known as TGN1412 [12]. The case represents a clear example of how an incorrect calculation of the dose and principle of action of the CD28 superagonist caused severe inflammatory reactions and chronic organ failure, instead of leading to an antitumor effect.

Therefore, on the one hand, we have a tumor that has gone beyond immune surveillance; on the other hand, we have an immune system that can provide an immune response if it is fed a portion of tumor neoantigens after the targeted destruction of the tumor. However, for this to happen, the immune response must be able to provide an abscopal effect (an impact on the entire body), but not cause immune system hyperstimulation, i.e., the amount of immunoactivators cannot be increased due to potential toxicity. The solution to this puzzle may come from the earliest attempts to use the immune system—the creation of vaccines. When the first similar problems related to the ineffectiveness of vaccines arose, the seemingly simple strategy of increasing the number of antigens to achieve the desired immune response often led to adverse reactions [13]. Pain and tenderness at the injection site were higher in vaccine recipients than those in placebo patients, and severity was clearly dose dependent [14]. For such antigens, the addition of an adjuvant made it possible to reduce the content of the antigen without compromising immunogenicity [13,15]. A similar principle can be applied to low immunogenic anticancer drugs, especially recombinant proteins.

Adjuvants are immunostimulatory molecules that trigger activations of innate and adaptive immune responses [16]. Immunoadjuvants are molecules that use tumors as antigen sources to provoke tumor-specific immune responses [17]. They resemble pathogen-associated molecular entities and interact mostly with Toll-like receptors (TLRs) on antigen-presenting cells (APCs), such as B cells, macrophages, and—most prominently—dendritic cells (DCs) [16]. The regulatory mechanism of adjuvants is related to the direct or indirect stimulation of APCs [18]. The maturation of DCs stimulated by adjuvants and the immune response to host antigens are two major prerequisites for therapeutic cancer vaccines to work [19]. DCs are an important messenger between CD4+ T helper cells and CD8+ T cells, and are associated with triggering further adaptive immune responses [20]. The mechanistic routes of adjuvants were summarized by Schijns: (1) the facilitation of antigen uptake transport and presentation by APCs, (2) the depot effect (prolonged antigen presentation), and (3) different signaling [21]. Classical inorganic adjuvants are the salts of various metals, which are substances that enhance or modulate the immune response to a vaccine [22]. Cancer vaccines are a new class of vaccines aimed at treating diseases rather than preventing infections, as is the case with conventional vaccines [23,24]. The modern embodiment of adjuvants are nanoparticles (NPs) that retain old properties (i.e., enhance the immune response) and acquire new, often specified, properties [25].

Nanomaterials are mainly exogenous and they can act as a danger signal when the innate immune system recognizes them [26,27]. “Danger signals” can be molecules that can be (1) DAMPs (damage-associated molecular patterns), which are produced by damaged cells in response to exposure to pathogens, toxins, mechanical damage, etc., as well as when inducing a strong immune response by activating the maturation of dendritic cells, and (2) PAMPs (pathogen-associated molecular patterns), which are sets of molecules associated with exogenous pathogens, such as bacterial lipopolysaccharides, endotoxins, peptidoglycan, as well as endogenous products of damaged and dead cells [28,29,30]. The interaction of these molecules with pattern recognition receptors (PRRs) elicits a response to “danger” within cells and in their microenvironment. The main PRRs are [31,32]:

- Agonists of Toll-like receptors (TLRs);
- NOD-like receptors (NLRs);
- Stimulator of interferon (IFN) genes (STINGs).

PAMPs can be used as adjuvants in the treatment of infections and other diseases because of their potential to enhance the immune response by combining with PRRs on antigen-presenting cells [33]. Metal-based nanomaterials are exogenous and exert an immune stimulation effect, which has led to the development of metal-based adjuvants for cancer immunotherapy [26,27].

Herein we collect and analyze the literature and the experimental data that indicate the possibility of using metal-based nanoparticles (MNPs) as adjuvants in cancer immunotherapy. Despite the long-standing use of adjuvants, their application has mostly been limited to infection disease vaccine development [29]. However, systemic research on the molecular mechanisms of action of adjuvants began not so long ago, as evidenced by the reviews of 2013 and 2017 [34,35]. Works on the role of MNPs as adjuvants in cancer immunotherapy are mostly fragmented. Most of these works focus on the immune responses induced by MNPs [27,30,36]. Only a few recent works casually consider MNPs as adjuvants [37,38]. This is not surprising, since most of the experimental studies addressing this issue have been published only recently (as is described further). The basis of successful antitumor therapy should be the abscopal effect, which represents a rare phenomenon in which the shrinkage of metastatic tumors occurs simultaneously with the shrinkage of a tumor receiving localized treatment [39]. Although its mechanism is still poorly understood, it has been proven that the abscopal effect is related to the immune mechanism [40]. We will discuss the conception of the cancer–immunity cycle [41], which has been reinterpreted by many researchers in favor of involving components of innate immunity. Metal-based NPs are inherently very well suited as innate immunity activators, being activators of PRRs whose role has been, in particular, actively studied in recent years. There is evidence that MNPs display adjuvant characteristics, promoting cell recruitment, antigen-presenting cell activation, cytokine production, and induction of a humoral immune response. MNPs could facilitate the induction of a cellular immune response, particularly T-helper 1 and T-helper 17, by functioning as adjuvants for subunit vaccines [35]. One of the features of the use of adjuvants is their intratumoral administration. This method of administration sometimes raises questions among researchers. For this reason, the possibilities of intratumoral administration are carefully considered in the examples of clinical trials (CTs). This is one of the most effective ways to reduce systemic drug toxicity. In some cases, MNPs have been the subject of CTs and have not caused serious side effects (see Section 3). We begin our rationale for the use of metal-containing NPs with a brief insight into ions and their immunomodulatory properties as precursors to the use of MNPs. In some cases, we found direct experimental data indicating the action of nanoparticles as an adjuvant in cancer immunotherapy. In other cases, there are only some prerequisites. However, we considered it important to indicate their presence so that readers can determine their importance for themselves. In conclusion, we combined the reasons in favor of the prospects for the development of combination drugs combining MNP as an adjuvant and another immunomodulator by applying the theory of superadditivity (see Section 5). We believe that our review contains the most relevant and up-to-date information about the possibility of using MNPs as adjuvants.

Thus, the main aim of this review is to outline the information about the prospects of using metal nanoparticles as adjuvants in the development of combined drugs, rather than try to cover all MNPs. We use a stepwise rationale to prove that the combination of metal nanoparticles with immunoactivators is very promising for the discovery of novel cancer immunotherapy drugs.

## 2. The Cancer–Immunity Cycle as a Target for Metal-Based Adjuvants

**Figure 1:**
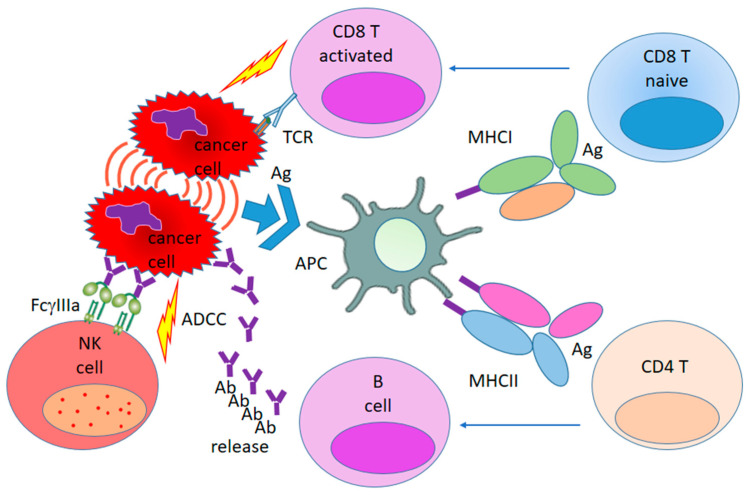
Augmented cancer–immunity cycle. The classic activation, through MHCI-CD8-acivated T cells, is on the upper side. The MHCII-CD4-NK axis is at the bottom. Ag—antigen, Ab—antibody, TCR—T cell receptor, and FcγIIIa—low affinity immunoglobulin gamma Fc region receptor III-A (CD16a).

The success of any cancer therapy relies on overcoming the immunosuppressive tumor microenvironment (TME), converting “cold” tumors into “hot” tumors, and therefore inducing a robust tumor-specific immune response that can kill cancer cells [42,43,44]. Thus, the intent of immunotherapy is to initiate or stimulate a self-sustaining antitumor immune cycle that will lead to persistent tumor regression. To attain an effective antitumor immune response, it is necessary to trigger a series of stepwise events, which Chen and Mellman combined into the concept of the cancer–immunity cycle [41]. At the beginning of the cycle, tumor-associated antigens are released as a result of the death of cancer cells. Further, antigen-presenting cells capture the released antigens, then process and present them in combination with MHC molecules to T-lymphocytes, resulting in the activation of immune cells. The activated cells migrate into the tumor and infiltrate it, which is where cancer cells are specifically recognized and killed. As a result of the death of tumor cells, a new portion of neoantigens are released; then, the cycle repeats, expanding its boundaries. The initial implication was focused on the presentation of tumor neoantigens to CD8 T cells, as well as the subsequent priming and activation of a pool of cytotoxic CD8 T cells. Further work in this area also showed the importance of the involvement of innate immune cells [45,46,47]. A simplified version of the cancer–immunity cycle that includes both adaptive and innate immunity is shown in Figure 1. Thus, the tumor antigens from cancer cells are captured by APCs. As exogenous antigens, tumor antigens that are endocytosed into the endosome–lysosome system usually bind MHC II molecules that are rich in the endosome, which further induce the priming and activation of CD4+ T cells. This pathway is that of classical humoral immunity, which kills cancer cells by antibody-antigen co-precipitation or antibody-dependent cell-mediated cytotoxicity (ADCC), which are mediated by NK cells (cited from [27]). Various components of the cancer–immunity cycle include both different elements of the TME and innate immunity cells [27,45].

In cancer patients, the immune cycle is inefficient, whereby cancer cells manage to implement the mechanisms of protection against the immune system [48,49]. Immune interactions in a cancer patient are wider than can be described by single steps and components; they comprise an emergent system in which exposure to individual agents can lead to unpredictable results [50], suggesting that such affectations should be carried out very carefully.

Thus, the ability to highlight some components that are active and cyclically expanding involving more and more new agents, being played into our hands. The involvement of innate immunity cells and, particularly, NK cells in response to a tumor makes it possible to consider this element of the immune cycle as an opportunity to enhance the entire cycle [46,51]. More interestingly, as we discussed in the introduction, the innate immune system itself can be activated by danger signals. Earlier in our work we hypothesized that the combined action of danger signals and an immunomodulator, which is issued from the same dying cancer cell within a limited space, would focus on a limited pool of immature dendritic cells, thus acting synergistically and enhancing their maturation and cytotoxic T-lymphocyte attraction potential [52]. At the same time, NPs are inherently exogenous molecules able to trigger the innate immunity. In simple words, the use of an adjuvant will at least have an effect due to this basic property of all particles, which can be enhanced by the features of the adjuvant used. This opens up great potential for cancer immunotherapy.

However, it is also imperative to physically deliver the necessary agents to the tumor and to initiate its physical death in order to destabilize the tumor homeostasis. The immunosuppressive TME is the main source of problems for triggering an effective immune response [37]. In addition, the physical barrier protects against the penetration of many drugs. The use of the silver bullet principle in its original meaning of hitting the monster’s heart can be reflected in the use of intratumoral (i/t) injection. Instead of trying to bypass the protective barriers of the tumor, we can deliver the drug to its very heart by intratumoral administration.

## 3. “Act Locally—Think Globally”

This expression is taken from [53], which outlines the basic principles of therapy that is conducted with local administration: by local administration of the drug, systemic toxicity is reduced while striving to achieve an abscopal effect of therapy by activating the immune system. The usual mode of delivery for immunomodulators is systemic because this route provides predictable pharmacokinetics and is considered to ensure full receptor occupancy in the tumor [54]. However, there is a problem in achieving therapeutic intratumoral concentrations in the absence of systemic toxicity, which is especially important for solid tumors [48]. Systemic toxicity that occurs during systemic administration often does not allow the use of optimal doses of drugs [55]. It is possible to reduce the toxicity inherent for immunotherapy by localizing a drug inside the tumor. Intratumoral delivery also offers the advantage of immediate agent access to tumor-draining lymph nodes, which are considered crucial sites for initiating and maintaining an antitumor immune response [53,56]. Due to many advantages—such as high local concentration, the possibility of the induction of the abscopal effect, and reduced off-target toxicity—intratumoral administration has been actively developed recently, as evidenced by the growing number of preclinical and clinical trials of drugs administered intratumorally. We have previously discussed this principle as applied to gene therapy [48], showing that the current development of gene therapy drugs may well follow this path. As applied to MNPs, this principle can be strengthened due to their innate properties, i.e., MNPs as exogenous agents, which activate the innate immune system through PRRs—in other words, acting as an adjuvant.

**Figure 2:**
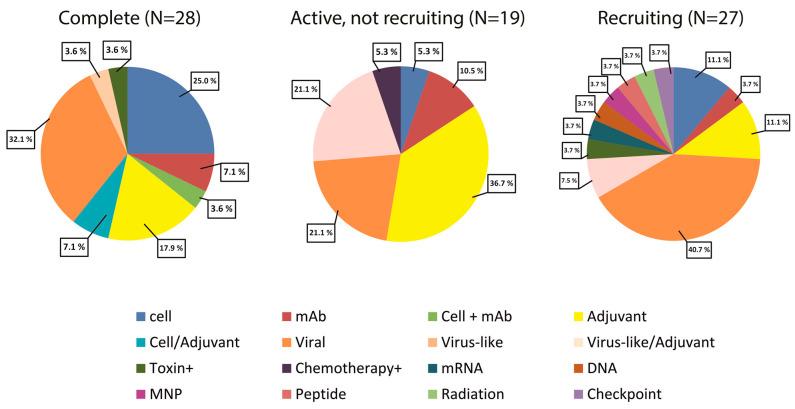
Clinical trials limited to “immunotherapy Intratumoral/Cancer”. An approximate distribution by type of therapy is given. See text for detailed explanations and limitations.

Since all the advantages of intratumoral administration in terms of basic principles to their use in gene therapy are considered in the above works [48,53], we will not repeat the previously analyzed examples. However, we will proceed to review data from clinical studies in which intratumoral administration was used. Therefore, at the moment, there are 818 studies in the Clinical Trials database (“https://clinicaltrials.gov/ (accessed on 19 February 2023”) for the query “Intratumoral/Cancer”. With the introduction of the restriction “immunotherapy intratumoral”, 135 studies can be found. We analyzed data from such studies in more detail, limiting the search to studies on recruitment status: Complete (31); Active, not recruiting (26); and Recruiting (42) (see Supplementary Table S1). Only studies that included intratumoral administration of a specific drug, not studies of intratumoral drugs or cell distributions, were selected. The results are presented in Figure 2.

As one can see, the number of studies in which the introduction of one or another compound directly into the tumor slightly decreased, amounting to 28, 19, and 27 studies, respectively. Most of these studies were represented by the introduction of various types of viruses into the tumor. This is not surprising given that the first FDA-approved intratumoral immunotherapy for cancer therapy was Imlygic (Talimogene laherparepvec, T-Vec)—an oncolytic herpes virus engineered to express human GM-CSF [57]. The next category of drugs that occupy a worthy place in intratumoral administration are certain TLR activators. They involve the danger signals system and, in fact, are adjuvants—substances that nonspecifically activate the immune system to achieve an antitumor immune response. Thus, the neoadjuvant Hiltonol (polyinosinic-polycytidylic acid stabilized with polylysine and carboxymethylcellulose, poly-ICLC) is a synthetic double-stranded viral RNA analog that mimics a danger signal by acting as a pattern recognition receptor agonist [58,59]. The intratumoral injection of poly-ICLC was well tolerated in patients with solid cancers and generated local and systemic immune responses, as was evident in the patients achieving clinical benefits [60] (See Clinical Trials NCT02423863, NCT03262103, and the combination of intratumoral Flt3L and Poly-ICLC with Low-Dose Radiotherapy (NCT01976585)). In addition, five studies (NCT04633278, NCT04698187, NCT04695977, NCT04916002, and NCT03983668) were based on CMP-001, a CpG-A oligonucleotide TLR9 agonist in a virus-like particle, which is rather difficult to categorize by type of action because in addition to DCs activation, it also activates an antitumor T cell response in an anti-Qβ antibody-dependent manner and leads to systemic antitumor T cell effects [61]. The other major part of therapies were cellular therapies based on the introduction of various cells, which were most often dendritic cells or cytotoxic T lymphocytes (CTLs) subjected to different modifications (refer to Supplementary Table S1).

Thus, intratumoral immune therapies that are in clinical trials can be roughly divided into (1) viral, (2) adjuvant, and (3) cellular. Of course, this is a very approximate distribution since oncolytic virotherapy combines both a response to a danger signal, according to its nature, and to lyse cells, triggering other immune processes. Cellular therapies can also have multidirectional effects depending on the modifications and type of cells administered. However, it is clear that the development of therapies based on intratumoral administration is a fairly rapidly developing area that is progressing very fast. The possibility of such therapies to influence metastasis can partly be estimated by the fact that about 3–4 dozens of clinical studies of intratumoral injections are performed on metastatic tumors, with subsequent evaluation of such therapy.

As for metal-based NPs, their presence in such types of CTs has not yet been noted; indeed, most of these studies date back to the last 2–4 years and represent preclinical studies (see below). We expect such drugs to appear in clinical trials in the near future. However, it cannot be said that metal-based NPs are absent in CTs. Therefore, in the clinical study NCT03589339, the drug NBTXR3, a novel radioenhancer composed of functionalized hafnium oxide crystalline NPs, was used. It has recently shown clinical activity in soft tissue sarcoma, hepatocellular carcinoma, head and neck squamous cell carcinoma, and advanced solid malignancies with lung or liver metastases. The patient received radiation twelve days after NBTXR3 injection. Daily CT-on-rails imaging demonstrated a retention of NBTXR3 within the tumor throughout the treatment period. At the initial follow-up evaluation, the lesion remained radiographically stable and the patient did not demonstrate treatment-related toxicity [62]. The best known drug based on metal NPs is Ferumoxytol—a superparamagnetic iron oxide NP with a polyglucose sorbitol carboxymethylether coating—which is commonly used for various anemias [63]. However, its use is not limited to this: it also turned out to be able to accumulate in tumors and is used for various tumor imaging systems [64]. A total of 30 studies were found for ferumoxytol/cancer (recruiting, not yet recruiting, active, not recruiting, completed, enrolling by invitation, and suspended studies). Moreover, its off-label use in intratumoral administration suggests that it has adjuvant properties (see Section 4.2).

Below, in this review, we will discuss the role of metal NPs/compounds for cancer immunotherapy. The features of intratumoral administration for cancer treatment and the possible effectiveness of this strategy will also be considered. The evidence to support local adjuvant therapy in immunotherapy with possible abscopal effects will also be reviewed.

## 4. To The Metals!

Before the advent of metal nanoparticles, metal ions were known for their roles in the normal functioning of the body. With the progression of research, it turned out that they are also strongly involved in various immune responses. Researchers continued to explore the functions of metal ions in cancer immunotherapy, and two new terms, “metalloimmunology” [65] and “cancer metalloimmunotherapy”, were described [66]. Metal ions participate in many cancer hallmarks, they influence the fate of cancer cells and participate in both innate and adaptive immunity by regulating hypoxia in the TME [67,68], thus inducing redox reactions with simultaneous free radical production [69,70], promoting innate immunity by enhancing the cytotoxicity of natural killer (NK) cells and the presentation capacity of DCs and macrophages [71,72], as well as by stimulating the activation of adaptive immune cells [73].

The important role of metal ion-based compounds as antitumor therapeutics is underlined by several pieces of evidence [74,75,76]. However, the variety of serious side effects, intrinsic and acquired drug resistance, and low target selectivity reduce the effectiveness of these agents, as well as greatly hampering their clinical use. Thus, the systemic application of metal ions may have toxic side effects [77]. Nanomedicine has the advantage of precise delivery and responsive release, which can perfectly overcome the drawbacks of metal ions, providing a breakthrough in the use of nanometallic materials in cancer immunotherapy [36].

Nanomedicine deals with synthetic particles on the nanometer scale, i.e., the dimensions of 1–100 nm [78,79,80]. As an ideal platform, NPs can deliver multiple drugs or integrate with other anticancer therapies (e.g., radiotherapy or immunotherapy) for combined or synergistic treatment strategies in addition to their own functions [81,82]. By targeting certain cells and tissues, NPs can boost the efficacy of immunotherapy, thus enhancing its immunostimulatory effect depending on its own characteristics, including co-localization, biodistribution, and release kinetics [83,84].

NPs possess the ability to accumulate at a tumor site because of the enhanced permeability and retention (EPR) effect of tumors (see Ref. [85] for an early overview of the pioneer works; [86]). Low lymphatic outflow from the tumor prolongs the residence time of the drug in it. The interaction of the NPs with the endothelium widens the existing gaps or induces new ones in the monolayer of vascular endothelial cells, thus increasing the access to the target sites in the organism. Such a structure in a tumor occurs due to increased nutritional requirements when a growing tumor creates a loose, chaotic capillary system with large pores between endothelial cells. This type of interaction can lead to NP-modulated endothelial leakiness (NanoEL) [87]. Meanwhile, some publications have shown that most nanomaterials enter tumor tissues via active transendothelial pathways [88]. Thus, the EPR effect alone cannot accumulate enough nanoparticles because of the high fluid pressure in the tumor stroma, irregular vascular distribution, and poor blood flow inside tumors [89,90]. This context challenges our current rationale for the development of cancer nanomedicines, but it suggests that an understanding of these pathways will unlock strategies for increasing tumor drug uptake [82].

Depending on the targeting properties of the nanomaterials, they can boost cancer immunotherapy in three different ways [91]: (i) targeting and killing cancer cells to induce immune-activating cell death (ICD) [92,93]; (ii) targeting the TME [94,95]; and (iii) targeting components of the peripheral immune system [96]. With the development of nanotechnology, different nanomaterials have been designed to enhance the curative potential of immunotherapeutic agents with more effective targeting of tumors and/or immune cells, as well as with reducing off-target adverse effects [97,98].

MNPs can perform completely different roles: (1) as a carrier, delivering different molecules to the tumor [99]; (2) as a functional agent, depending on physical and chemical properties — acting as a photothermal vaccine [100], photodynamic activator [101], magnetic guidance [102], etc.; and (3) as an immunomodulator [103].

MNPs play a major role in nanotechnology and nanoscience, and have found several applications in the medical, biomedical, and pharmaceutical area of nanotechnology for their magnetic [104,105], thermal [106,107], electronic, and optical properties [108,109]. Compared to non-metallic nanoformulations of similar sizes, the higher density MNPs are more readily taken up by cells, providing a benefit for different cancer therapy strategies [110,111]. It is very significant that MNPs are flexible in being modified with diverse functional groups, which thus permits them to associate with drugs of interest, ligands, antibodies, etc. Such properties represent particular advantages in cancer immunotherapy applications [112,113], due to the flexibility and precision with which their characteristics can be controlled [114].

Herein, the mechanisms of action of MNPs can be summarized as follows:

- PRRs activation;
- RedOx dyshomeostasis;
- Modulation of its related biological signaling pathways.

Considering the role of metals as immunostimulators, the applications of MNPs in the context of immune system modulation represent a currently exciting new area. Therefore, understanding the interaction of the innate and adaptive immune systems with MNPs is a crucial step in expanding the knowledge to improve the efficacy of immunotherapy and to ameliorate cancer treatment strategies.

The interplay of ions, NPs, and metal-based compounds is a hot topic for discussion [36,115]. However, it is out of our scope, so we will take into account any study that reports metals, in any embodiment, acting as adjuvants. MNPs act as immunostimulants by promoting the activation and maturation of immune cells. Several strategies have included MNPs as vaccine platforms, such as using the MNPs of different materials (including gold, iron oxide, and nickel), shapes (including spheres, cubes, rods, and disks), sizes (from 2 nm to over 200 nm), and types of coating [35]. The U.S. Food and Drug Administration also refers to these materials as “materials that have at least one dimension in the range of approximately 1 to 100 nm and exhibit dimension-dependent phenomena” [116]. MNPs are relatively non-biodegradable, have a rigid structure, and possess simple synthesis methodology. Many of them have been studied for their immunological properties [117]. Next, we will consider the possibilities of using MNPs, referring to specific examples and supporting data, in activating the immune system and acting as adjuvants.

### 4.1. Alum

The immune-enhancing effect of aluminum salts (alum) was first reported in the 1920s, which is when it was found that the injection of potassium-aluminum-precipitated diphtheria toxoid into guinea pigs provided a greater protection than toxoid alone [118,119]. Since then, alum-containing adjuvants have been used in billions of vaccine doses and, until recently, represented the only adjuvant approved in the United States [29,120]. Alum is the only widely used human adjuvant, and it has been used for more than 100 years due to its minimal reactogenicity and low cost. The degree of antigen adsorption by aluminum-containing adjuvants is considered an important characteristic of vaccines that are related to immunopotentiation by the adjuvant. The adsorption of antigens onto alum increases uptake, stability at the site of injection with prolonged effect, and the stimulation of the immune system [121,122,123]. Different models that included non-adsorbed antigens in the vaccine formulation induced higher antibody titers than those that were induced by either a solution of antigen without adjuvants or with adsorbed antigens by an aluminum phosphate adjuvant [124]. Aluminum adjuvants appear to enhance the immune response via several molecular pathways, support the activation of CD8 T cells, and selectively stimulate a Th2 immune response upon injection [125].

The adjuvanticity of alum comes from its ability to induce a local pro-inflammatory reaction and to enhance the immunogenicity of a wide range of soluble antigens [126]. For example, heterogeneous micron particles of aluminum hydroxide aggregates could be recognized and phagocytosed by macrophages or dendritic cells [127]. Preclinical studies have demonstrated that aluminum nanoparticles (Al-NPs) are potential adjuvants for developing vaccines for the purpose of cancer immunotherapy [128,129,130]. Al-NPs (nanosized AlO(OH)) can deliver antigens and adjuvants to antigen-presenting cells in lymph nodes and thereby augment antigen-specific CD8+ T cell responses, as well as promote Th1 type immunity, which is in contrast to the Th2 immunity promoted by the traditional aluminum adjuvant [131,132].

In some studies, FDA-approved adjuvants containing Al (Alhydrogel^®^ (InvivoGen, San Diego, CA, USA) and Adju-Phos^®^ (InvivoGen, USA)) were used to prepare the nanosized particles. As NPs can reaggregate quickly (from 10 h to 14 days, respectively), studies were conducted to stabilize these NPs. Orr et al. stabilized rod-shaped Al(OH)3-NPs (~60 nm) from Alhydrogel^®^ using an anionic polymer and polyacrylic acid [130]. Vrieling et al. prepared ~200–400 nm AlPO4-NPs by sonicating Adju-Phos^®^ [133].

The green synthesis of aluminum-integrated zeolitic imidazolate nano-metal–organic frameworks (MOF) was carried out by the team of Zhong X et al. in order to co-deliver antigens and adjuvants by taking advantage of a natural biomimetic mineralization process. Aluminum-adjuvant-integrated nano-MOF can enhance humoral and cytotoxic T lymphocyte immune responses against cancer. Aluminum-integrated MOF after subcutaneous injection were accumulated in the lymph nodes and taken up by dendritic cells in immunized mice [134].

The biorxiv preprints propose the use of aluminum hydroxide adjuvants to retain drugs in brain tumors, which are difficult to use for immune impacts with local precision [135]. In mouse models of both immunologically hot and cold brain tumors, the intracranial deposition of alum-tethered cytokines has been shown to cause significant delay in tumor progression vs. alum alone. Intracranially injected alum-tethered mouse serum albumin (MSA) fused to IL-2 (MSA-IL-2) and IL-12 cause a potent antitumor immune response. They engage both innate and adaptive immune mechanisms, as well as preserve neuronal function and dynamics by promoting the immune surveillance of tumors. The brain tissue of mice carrying tumors—treated with cytokines bound to alum—was enriched by the expression of genes consistent with healthy brain function, including genes involved in carbohydrate or lipid metabolism, which are similar to the brain tissue from a naïve subject [135].

Last year, two papers were published, under the supervision of Prof. Wittrup, devoted to the possibility of the intratumoral administration of NPs based on aluminum hydroxide (alum) and recombinant cytokines. A chimeric cytokine containing an alum-binding peptide (ABP) tagged to the cytokine has been shown to bind to alum via ligand exchange between hydroxyls on the surface of the alum and phosphoserine residues of ABP. Such particles are able to retain cytokines in tumors for weeks-long time with minimal side effects.

A single dose of alum-tethered interleukin-12 induced substantial interferon-γ-mediated T-cells and natural-killer-cell activities in murine melanoma tumors, as well as increased tumor antigen accumulation in draining lymph nodes and elicited robust tumor-specific T-cell priming. The intratumoral injection of alum-anchored cytokines enhanced responses to checkpoint blockade, and promoted cures in distinct poorly immunogenic syngeneic tumor models, as well as elicited control over metastases and distant untreated lesions. Thus, the alum-bound IL-12–ABP and IL-12–ABP were administered with an intratumoral injection in subcutaneously (s/c) implanted B16F10 melanoma tumors. The IVIS whole-animal fluorescence imaging of labeled alum-bound IL-12–ABP showed a persistence of the cytokine at high levels in the injected tumors for weeks after a single dosing; meanwhile, the signal from IL-12–ABP that was injected without alum was rapidly cleared [136]. The i/t injection of free IL-12 led to animal weight loss, significant elevations in serum IFN-γ and alanine transaminase (ALT, indicating liver toxicity), and reduced albumin and total protein levels in the blood after a single dose. By contrast, the i/t injection of alum-IL-12–ABP-p elicited significantly lower serum IFN-γ levels, prevented ALT levels from exceeding the normal clinical range, and left blood chemistry unaffected, thus correlating to no weight loss in the treated animals. Thus, alum binding to cytokine alters its biodistribution profile and makes it local and safe. The antitumor efficacy was concordantly increased: a single i/t dose of alum-IL-12–ABP led to complete responses in 11 of 13 animals in the Ag104A tumor model, while the unanchored IL-12–ABP was only moderately effective and led to treatment-related mortality in 1 of 13 animals. However, the clinical success of any intratumoral therapy depends on its ability to stimulate the systemic antitumor responses in order to control distal, untreated lesions and micrometastases, i.e., the presence of an abscopal effect [137]. As it was shown in the bilateral tumor model, a single i/t injection of alum-IL-12–ABP into one tumor may lead to the shrinkage of tumors in the opposite flank of mice [136]. Meanwhile, in other mice models, the scenario of neoadjuvant therapy was confirmed. Thus, a single dose of alum-IL-12 elicited a systemic immunity enabling control over non-injected distal tumors and confirmed the possibility of the abscopal effect in an i/t cancer immunotherapy approach.

In the second study, the intratumoral administration of two IFN I subtypes (IFNa, IFNb) at different drug retention times in tumors were compared [138]. The intratumoral retention of type I interferons significantly improves the treatment efficacy and reduces toxicity. Alum-ABP-IFNα significantly extended the survival over IFNα, and alum-ABP-IFNβ was more efficacious than IFNβ for MC38 and B16F10 tumor models. Type I intratumoral IFNs have also been effective in combination immunotherapy. Almost complete cure rates were achieved when intraperitoneal (i/p) IL2 was combined with alum-ABP-IFNα (6/6 cures) or alum-ABP-IFNβ (6/7 cures), while no mice were cured when i/p IL2 was combined with IFNα or IFNβ. Similar results were obtained for the combination of i/p αPD1 and i/t alum-ABP-IFNα or alum-ABP-IFNβ. However, combined immunotherapies result in contrasting memory responses. A poor resistance to rechallenge in mice cured by IL2 combined with alum-ABP-IFNα (1/6 survivors) or alum-ABP-IFNβ (1/6 survivors) was observed [138]. Mice cured with αPD1 + alum-ABP-IFNβ also had poor resistance to rechallenge (1/4 survivors). In contrast, a strong resistance to rechallenge in mice cured by αPD1 combined with alum + ABP-IFNα (5/6 survivors) was also observed. Therefore, intratumoral retention is a promising strategy for type I IFN, but requires careful consideration of the tumor phenotype and combination therapy agents.

In the last few examples, one can see how the modern approach to drug design has elegantly solved the problem of the complication of retaining therapeutic proteins in the tumor tissue. It was demonstrated how the use of aluminum NPs has helped solve this problem by acting as an anchor for the protein.

### 4.2. Ferrum

**Figure 3:**
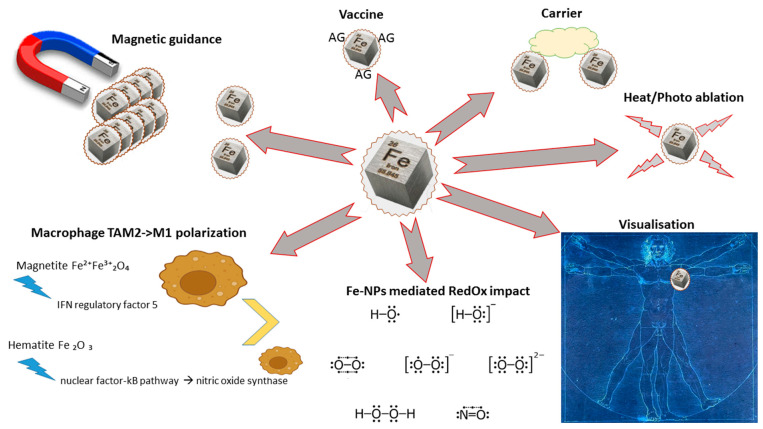
Possible applications of ferrum-based NPs. Fe—a ferrum-based NP and AG—antigen.

The development of magnetic NPs (MagneticNPs) as delivery systems has opened up the possibility of using local enhancement methods, so that the specific effects of MagneticNPs and/or the delivered drug can be elicited in a determined area [139]. Iron oxide as magnetic NPs (IONPs) has shown promising results in preclinical and clinical settings for numerous applications, including oncology. Possible applications for oncology are summarized in Figure 3 and in some reviews (for instance [140,141]). IONPs have been used to improve the efficacy of current immunotherapeutic approaches in in vivo studies because they are biocompatible, biodegradable, and do not pose long-term toxicity concerns regarding their degradation products. Moreover, their surface can be tailored to link drugs, antibodies, and homing ligands in order to target specific cells [142]. One of the forerunners of local delivery can be considered direct or indirect magnet-driven intratumoral delivery, allowing the iron NPs’ localization at the tumor site [143]. The use of IONPs in adoptive cell therapies and T-cell enrichments in targeting the delivery of magnetic cytotoxic T cells and NK cells to specific tumor sites was also explored [142,144]. This strategic approach can attenuate the side effects in the body associated with T-cell exposure, and it requires only a small amount of T cells to obtain therapeutic effectiveness. Although, this is an under-explored field, some encouraging preliminary results have been achieved, such as in the in vitro migration of T cells driven by external magnetic fields and the increased retention of IONP-labeled CD4+ and CD8+ cells in the popliteal lymph node when compared with non-magnetic T cells [145,146]. An even greater challenge is in being able to improve NK-cell-based therapy using IONPs, which typically involve the dispersion of NK cells upon in vivo administration and limited infiltrations into tumor environments. Similar to magnetic T cells, preliminary results showed that the function of NK cells was not impaired; they migrated in response to the magnetic field and, in the in vivo experiment, the injection of magnetic NK cells resulted in the greatest inhibition of tumor growth due to an increased retention at the tumor site [147,148].

IONPs are able to modulate antitumor immune-response reprogramming tumor-associated macrophages (TAMs) in the TME into pro-inflammatory M1 phenotypes through iron accumulation inside the cells [149]. Recently, it was demonstrated that the use of off-label ferumoxytol (Fe_3_O_4_) inhibited mammary tumor growth and lung cancer metastases. Ferumoxytol-treated macrophages displayed a higher level of pro-inflammation Th1-type mRNA and M1 macrophage polarization. After incubation with ferumoxytol and macrophages, adenocarcinoma cells showed higher caspase-3 activity for tumor cell killing [150]. It was also shown that the subcutaneous injection of IONPs into the mice mammary fat pads alongside MMTV-PyMT-derived cancer cells revealed a significant suppression of the breast tumor growth associated with an increased presence of pro-inflammatory M1 macrophages in tumor tissues. In addition, the intravenous administration of ferumoxytol after tumor cell injections significantly reduced liver and pulmonary metastasis; whereas, before the injection of tumor cells, it prevented the development of liver metastasis, suggesting the potential role of IONPs in addressing metastasis outgrowth and altered macrophage polarization in hepatic metastasis [150]. There may be differences in macrophage activation by iron oxide: it was found that magnetite-induced macrophage activation and M1 polarization via the activation of the IFN regulatory factor 5, was more effective—than the activation by hematite through the nuclear factor-kB pathway [151].

Ferumoxytol has been shown to be effective in a murine leukemia model and in patient-derived xenotransplants bearing leukemia cells expressing low levels of ferroportin, which is an iron efflux transporter. In vitro intracellular iron overload has been responsible, most likely via ferroptosis, for a massive increase in ROS and cell death. The intravenous administration of ferumoxytol at 6 mg/kg in sub-clinical therapeutic doses for anemia (7.3 mg/kg) reduced leukemia burden, improved overall survival, and was able to selectively target patient-derived xenograft (PDX) leukemia cells with low FPN without harming normal cells [152].

An intriguing study carried out by Chin et al. showed that the intravesical instillation of Fe_3_O_4_@Chl/Fe CNPs, modified with 4-carboxyphenylboronic acid (CPBA), targeted and induced the local killing of bladder cancer cells by promoting singlet oxygen production and ferroptosis through a combined photodynamic and chemodynamic therapeutic strategy. One of the findings of this novel theranostic approach was the reprogramming of TME, which shifted from an immunosuppressive to immunostimulatory effect. This occurred with a reduction in PD-L1, IDO-1, TGF-β, and M2-like macrophages, as well as with the induction of CD8+ T cells and the increase in survival rates from 0 to 91.7% [153].

A modern and recent approach for immunotherapy in cancer aims to enhance both humoral and cellular immune responses in the tumor site, avoiding adverse systemic effects. For this, NPs must have adjuvant properties, as stated above. Indeed, among IONP applications, iron NPs are also used as adjuvants for vaccines because they can enhance the immune response against pathogens, or in cancer cells modulating the function of the host immune system in several manners, including complement system activation, immune cell recruitment, and inflammasome activation [154]. The administration of low doses of iron NPs induces an adjuvant outcome in the Th2 response in mice [155]. The subunit of the *Mycobacterium tuberculosis* vaccine with IONPs as an adjuvant enhanced the response of specific Th1 (CD4+ IFN- γ), Th17 (CD4+IL-17+), and TCD8+ cells [156].

Regarding cancer vaccines, the effect of IONPs has been investigated in several studies. In a study performed by Grippin et al. on cancer vaccines, based on the delivery of tumor antigens by IONPs, it was demonstrated that IO-RNA-NPs enhanced ex vivo DC activation, stimulating the expression of costimulatory markers (CD80, CD86, CD40), as well as the secretion of IFN-α when compared to electroporation. As a model for adoptive cellular therapy, bone marrow-derived dendritic cells (BMDCs) treated with IO-OVA RNA-NPs and incubated with naïve OVA-specific OT1 T-cells or antigen-experienced OVA T-cells significantly enhanced the activation of antigen-experienced T-cells and the priming of naïve T-cells in an antigen-specific manner. When the DCs loaded with IO-RNA-NPs were injected intradermally, the migration to lymph nodes was enhanced, and it significantly inhibited the growth of subcutaneous B16F10-OVA tumors. Moreover, the application of an MRI technique enabled quantitative DC tracking and the MRI-detected DC migration at two days post-vaccine, which predicted the antitumor efficacy of the therapeutic DC vaccine [157].

To reveal the adjuvant properties of Fe_3_O_4_ NPs, a study was conducted on a mouse model of H22 liver tumors. Fe_3_O_4_ NPs have been compared with alum adjuvants for their ability to induce immunity in suppressing tumor growth rate in prophylactic and therapeutic studies. The results showed that the Fe_3_O_4_ NPs adsorbed by the autovaccine have a greater advantage over alum-autovaccine adjuvants after subcutaneous injection. They also increased the tumor mass suppression and cytotoxic activity of T lymphocytes, and induced a higher level of IFN-gamma [158]. As a proposed platform for cancer vaccine with adjuvant properties, the superparamagnetic Fe_3_O_4_ NPs combined with ovalbumin have been found to be safe and effective in stimulating immune responses after intratumoral administration in BALB/c mice that were injected with CT26 cell suspensions. Fe_3_O_4_-OVA NPs markedly inhibited tumor growth and induced potent humoral and cellular immune responses by promoting the activation of immune cells and cytokine production [159]. An intratumoral injection of ferumoxytol combined with an alternating magnetic field (AMF) produced a ferumoxytol dose-dependent tumor killing, as well as histology visualization of ferumoxytol distribution near the tumor periphery confirmed the spatial correlation of cell death with ferumoxytol distribution [160].

As can be seen, the iron-based NPs can activate the immune responses in various ways, including as adjuvants. The intratumoral administration of such particles has not yet shown serious side effects, and, given the widespread use of ferumoxytol for tumor imaging in clinical studies, such particles can be considered safe to a certain extent. It is worth noting that experimental data suggest that the adjuvant properties of iron-based particles may be stronger than those of alum-based particles. This may be of interest in future research.

### 4.3. Titanium

Titanium dioxide NPs (TiO_2_NPs) have a safe profile and are nontoxic to mammalian cells as they are chemically inert and stable under physiological conditions [161,162,163]. This biocompatibility has attracted great interest and has made it possible for its use in numerous applications, ranging from pigments in paints, toothpaste, the absorption of ultraviolet light in sunscreen lotions, food additives, and—recently—agriculture and biomedicine [164].

Unlike IONPs, the nanobiomaterials based on TiO_2_ NPs have been less studied for cancer therapy; however, their potential for the delivery of anticancer drugs and genes, as well as their effect as photosensitizers for photodynamic therapy due to their photocatalytic characteristics have been mainly addressed [165,166,167,168].

The properties of titanium NPs (TiNPs) have been extensively studied in the context of oncology, particularly in light of the latest theranostic approaches, which explore the intratumoral localization of titanium NPs for enhancing the effects of imaging-guided radio- or sonodynamic therapy [169,170]. However, the antitumor effects induced per se by locally administered TiNPs alone have been barely investigated. A 2015 study reported the possible in vivo antitumor application of different formulations of TiO_2_NPs in CT26 subcutaneous tumor-bearing mice. The intratumoral insertion of a TiO_2_ gel plug inhibited tumor growth, with a probable induction of oxidative stress in a size-, dose-, and time-dependent manner. This route of administration exerted a low host toxicity when compared with intraperitoneally administered TiO_2_NP gels or with the systemic (subcutaneous or intraperitoneal) administration of TiO_2_NPs suspended in PBS [171].

In the field of adoptive T-cell therapy for the treatment of cancer, NPs are proposed in order to overcome many hurdles, mainly addressing insufficient T-cell trafficking and the suppressive tumor microenvironment. Particularly, TiNPs with nickel, commonly known as nitinol, were developed as micromesh implants for loading with tumor-specific human chimeric antigen receptor (CAR)-T cells in order to improve the treatment of solid tumors with T-cell therapy. In a preclinical mouse model of human pancreatic cancer, expressing the tyrosine kinase-like orphan receptor (ROR1), a device with high CAR-T cells loading was implanted directly to the tumor surfaces. There, it fostered the rapid expansion of T cells and the delivery of a high amount of T cells directly into the tumor site without CAR T-cell exhaustion. It was also shown that CAR-T-cell-loaded thin films inhibited solid tumor growth, improving animal survival with respect to intravenous or intratumoral T-cell delivery [172].

**Figure 4:**
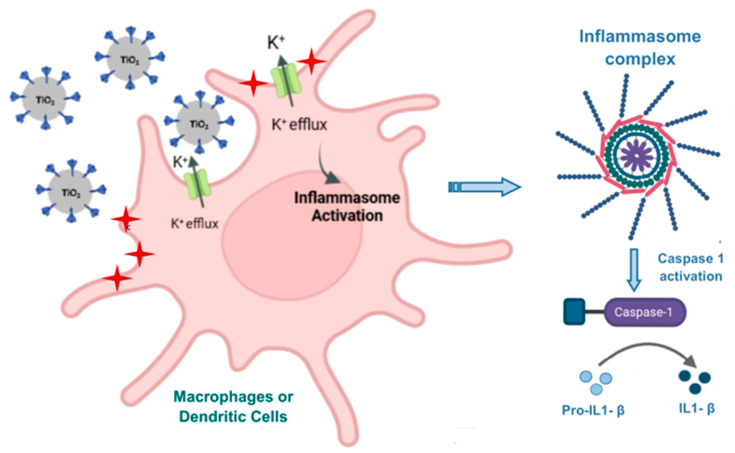
Possible mechanism of the triggered immune cells and the immune response amplification via the K+ mediated-inflammasome activation by functionalized TiO_2_ nanoparticles.

Some studies have reported the modulation of immune responses by titanium. One of these studies exclusively investigated in vitro the immunomodulatory effects of TiO_2_ derivatives on mixed lymphocyte reaction cultures, which is a model for measuring the proliferative responses of allogeneic responder lymphocytes. This study revealed that the nanostructural materials suppressed splenocyte proliferation via a mechanism related to reduction in IL-2 and IFN-γ secretion by Th1-type cells, thus demonstrating that TiO_2_ based-nanostructural materials have immunomodulatory properties. These properties likely block T-cell-mediated responses in vitro; therefore, one might find an application for the treatment of graft versus host diseases and autoimmune disorders [164]. Other in vitro studies reported an increased expression of a variety of pro-inflammatory cytokines in murine macrophages (RAW 246.7), as well as in murine dendritic cells (bm-DC) [173]. It was also shown that the airborne exposure to TiO_2_NPs could induce respiratory allergies through a possible adjuvant-like activity of NPs on an allergic sensitization that is associated with the impairment of the immune response [174,175]. All these effects on the immune system can be associated with the physicochemical properties of TiO_2_-based particles, which can affect their recognition and uptake by immune cells, such as in macrophages, monocytes, platelets, leukocytes, and dendritic cells; moreover, these can subsequently trigger an inflammatory response. For instance, TiO_2_ microparticles decorated with nanospikes resemble the spike-like nanostructures found on the surface of pathogens and were able to activate the innate immune response. They were actively uptaken by macrophages and activated inflammasomes via the K+ efflux from DCs during phagocytosis (see Figure 4). As a result of the mechanical stress induced by the spiky particles, the inflammasomes are activated and subsequently the innate immunity was stimulated [176]. The inflammasome activation by spiky particles combined with monophosphoryl lipid A (MPL), an agonist of TLR4, also promoted in vitro and in vivo DCs maturation, which was characterized by CD40 upregulation and is critical for the subsequent activation of CD8+ cytotoxic T-cell responses against intracellular pathogens and cancer cells. Indeed, the immunization of mice with OVA occurred together with spiky + MPL exerted OVA-specific CD8+ and CD4+ T responses. A similar trend was observed in mice immunized to the 2009 H1N1 influenza vaccine with an intramuscular injection combined with spiky + MPL when compared to the MF59-like adjuvant (AddaVax), aluminum hydroxide (alum), and the other tested combinations [176].

The previously described study on spike TiO_2_ also evidenced the adjuvant effects of these in vivo titanium structures on mice that were subcutaneously inoculated with EG7 tumor cells. The s/c injection of spiky + MPL-activated DCs almost completely inhibited tumor growth, indicating the efficacy of DC-mediated cancer immunotherapy. The effects of TiO_2_-nanorods (NRs) on the immune function and their potential application in immunotherapy was performed by Wang et al., who demonstrated that TiO_2_-NRs exhibited immunomodulatory effects on macrophage and lymphocytes, and also stimulated the key antitumor cytokines TNF- and IL-2 in a time- and concentration-dependent manner [177]. In an interesting work, You et al. explored the effects of long-circulating hydrophilized titanium dioxide NPs(HTiO_2_ NPs) in SCC7 tumor-bearing mice. They demonstrated a high tumor targetability 24 h after intravenous administration, indicating a selective accumulation of HTiO_2_ NPs in the tumor tissue. Upon the ultrasound activation (SDT) of HTiO_2_ NPs, they generated, as sensitizers, reactive oxygen species that caused the growth suppression of superficial tumors and also destroyed the microvasculature. This strategy also showed antitumor efficacy against deep liver tumors with substantial suppressions of tumor growth. A macroscopic analysis showed no signs of metastasis in the main organs of animals treated with HTiO_2_ NPs-SDT. These results were associated with an immunomodulant effect characterized by enhanced intratumoral immune responses with high levels of certain chemoattractants, such as IL-6, IL-1β, and tumor necrosis factor-α. However, no change was detected in their circulating levels, suggesting that HTiO_2_ NPs with SDT could be used as an adjuvant therapy [178].

Thus, TiNPs, on the one hand, have the ability to act as an adjuvant in immunotherapy. On the other hand, some of their properties can make them dangerous, such as acting as allergens, affecting vascular integrity, or in promoting cancer cell intravasation and extravasation, as well as in inducing metastasis in murine breast cancer models [179].

### 4.4. Manganese

Manganese oxide (MnO_2_) nanomaterials have a stable structure, as well as excellent physical and chemical characteristics. Lin and co-authors described the mechanism of oxygen generation by MnO_2_ NPs inside the TME (hypoxia) [180]. MnO_2_ depletes reduced glutathione (GSH) to an oxidized form (GSSG); in addition, it produces Mn^2+^ ions through a redox reaction. These Mn^2+^ ions react with the intracellular H_2_O_2_ and bicarbonate to produce hydroxyl radicals, which enhance the intracellular production of toxic hydroxyl radicals (·OH). Therefore, Mn^2+^-mediated GSH depletion prevents OH radical scavenging. In 2017, Yang et al. developed a nanoplatform based on hollow manganese dioxide (H-MnO_2_) nanoshells, which is a multifunctional theranostic platform for promoting anti-tumor immunity that can advance cancer immunotherapy. These nanoplatforms are able to modulate the tissue microenvironment, and they can release chlorine-e6 with doxorubicin, as well as inhibit tumor growth alone or in combination with programmed death-ligand 1 (PD-L1) checkpoint blockades. Upon accumulation in the tumor, the acidic pH of the TME triggers the decomposition of MnO_2_ to Mn^2+^. In addition, it can also induce a reaction with H_2_O_2_ to form water and oxygen, thus consuming GSH, which can react with ROS within the TME, favor the antitumor immune responses, and serve as enhancers for photodynamic therapy-induced (PDT) immunotherapy and photothermal agents (PTAs). Although the dual combination of PDT and chemotherapy with H-MnO_2_ exerted a synergistic antitumor effect, tumors continued to grow, albeit at a slower rate than the control group [181]. A hollow MnO_2_ nanodrug delivery system was able to enhance antitumor effects, with a primary distribution in tumors.

Mn@CaCO_3_/ICG NPs (MnO_2_ NPs covered with CaCO_3_ and indocyanine green) loaded with PD-L1-targeting siRNA were developed as an integrated nanoplatform, which combined PDT with immunotherapy to enhance photodynamic therapeutic effects and simultaneously inhibited tumor cells resistance/evasion. In vivo experiments demonstrated that the nanoplatform could efficiently release uploaded PD-L1-targeting siRNA to the tumor tissues and could also significantly improve tumor hypoxia, which further contributed to the therapeutic effect of PDT in vivo [182].

To underline the importance of a combination of diverse inducers for the synergistic effect of cancer immunotherapy, a study by Sun et al. proposed tadpole-ovoid manganese-doped hollow mesoporous-silica-coated gold nanoparticles (Au@HMnMSNs) as biodegradable catalytic cascade nanoreactors, which were constructed to generate intratumoral high-toxic hydroxyl radicals combined with DOX and Aspirin (ASA) for the purposes of enhancing the induction of ICD and for the maturation of DCs [183]. PEG–Au@HMnMSNs can release Mn^2+^, inducing a Fenton-like reaction thus producing •OH through the degradation of GSH in TME. In addition, it can protect the glucose oxidase GOx-like activity of Au, thus regenerating H_2_O_2_ for an enhanced chemodynamic therapy (CDT) effect. Such an inherent GOx-like catalysis-enhanced CDT combined with a DOX treatment could induce sufficient ICD in order to promote DCs maturation, which can thus enhance the intratumoral infiltration of CTLs when compared with monotherapy.

**Figure 5:**
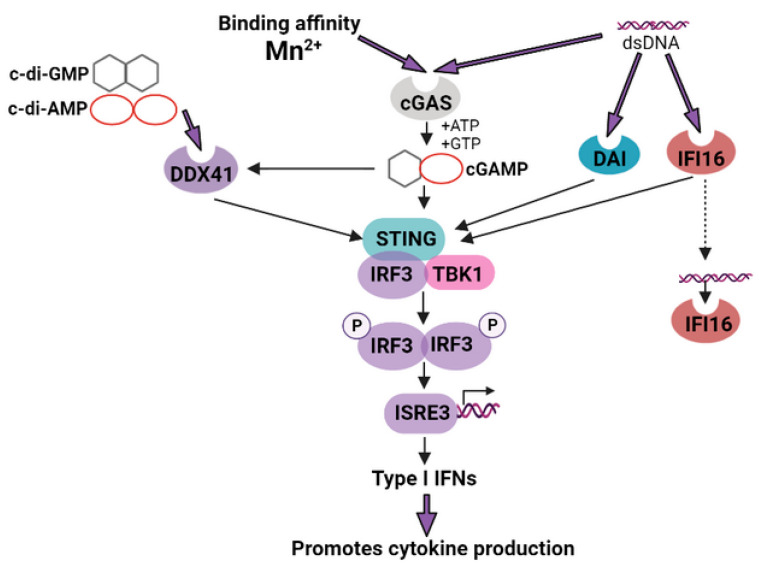
Mn^2+^ as an activator of the cGAS-STING pathway. Abbreviations: cGAS—cGAMP synthase; IFI16—interferon γ-inducible protein 16; DDX41—helicase DEAD box polypeptide 41; c-di-GMP—cyclic di-GMP; c-di-AMP—cyclic di-AMP; STING—stimulator of interferon genes; IRF3—interferon (IFN) regulatory factor 3; TBK1—TANK-binding kinase 1; cGAMP—cyclic guanosine monophosphate-adenosine monophosphate; DAI—DNA-dependent activator of IRFs; IFNs—type-I interferons; STING (also known as MITA, MPYS, or ERIS); cGAMP—cyclic GMP-AMP; and IRF3—IFN-regulatory factor 3.

Mn^2+^ have recently been shown to be activators of the cGAS-STING pathway [73,184] (see Figure 5). It has been proven to play critical roles in the initiation of cancer immunity, ameliorating the immunosuppressive network in “cold” tumors [185]. Amorphous porous manganese phosphate (APMP) NPs that were highly responsive to TME were employed to constructs of doxorubicin (DOX)-loaded and phospholipid (PL)-coated hybrid NPs (PL/APMP-DOX NPs). This triggered the release of DOX, which induced DNA damage, and also caused Mn^2+^ to augment cGAS/STING activity. These nanoactivators, injected subcutaneously, were able to boost dendritic cell maturation and increased cytotoxic T lymphocyte infiltration in the tumor site [103].

Recently, Wang et al. revealed that Mn^2+^ was required for the host defense against DNA viruses, which was achieved by increasing the sensitivity of the DNA sensor cGAS, enhancing the activation of the cGAS-STING signaling axis, and in increasing the STING-cGAMP binding affinity [186]. In some cases of advanced metastatic solid tumors, Mn^2+^ increases the clinical efficiency of cancer treatments such as PD-1/PD-L1 therapy, thus improving prognosis [73]. Considering the indispensable role of Mn for the host defense against cytosolic dsDNA, achieved by activating cGAS-STING, Mengze et al. used Mn-insufficient mice with greatly reduced tumor-infiltrating CD8+ T cells in order to show significantly enhanced tumor growth and metastasis. A synergic effect has been shown by combining the intraperitoneal treatment of Mn^2+^ and immune checkpoint inhibition; this process boosts antitumor efficacies and reduces the anti-PD-1 antibody dosage required in mice. Importantly, a completed phase 1 clinical trial with the combined regimen of Mn^2+^ and anti-PD-1 antibodies showed promising efficacy [73].

Additionally, a CpG-oligodeoxynucleotide-coated Mn-phenolic network of NPs, as an effective immune activator, can effectively internalize into macrophages and stimulate M1 polarization in order to promote the release of proinflammatory cytokines. This simultaneous treatment successfully inhibits tumor growth and prolongs survival in a mouse tumor model [187]. Meanwhile, other authors have shown that Mn^2+^ does not induce the production of the proinflammatory cytokines IL-1 and IL-18 in humans nor in mice, even though they stimulated a similar production of IL-6 and TNFα [188].

Studies from Sun et al. showed a prototype of cancer metallo-immunotherapy by using STING agonists, cyclic dinucleotide (CDN) stimulators of interferon genes, and Mn^2+^. They reported that Mn^2+^ markedly increased the type-I IFN activities of STING agonists in multiple human STING haplotypes and that Mn^2+^ self-assembled with CDN STING agonists to form a coordination NP (CDN-Mn^2+^ Particle, CMP), which elicited robust antitumor immunity after local or systemic administration, indicating a promising novel platform for metallo-immunotherapy [66]. Some authors suggest that soluble Mn^2+^ is unable to induce a local immune response with respect to the Mn^2+^ that forms particles in PBS. Using jelly-like Mn^2+^ colloids (MnJ, Mn Jelly) consisting of elongate NPs, authors have developed a stable MnJ without aggregation: the adjuvant effect is retained after storage at −80 °C for three weeks, or even after the freeze–drying/lyophilization cycle. This represents an important advantage over aluminum vaccines, which are very sensitive to freezing and are hard to store or transport. A colloidal manganese salt (Mn jelly, MnJ) was formulated to act not only as an immune potentiator, but also as a delivery system to stimulate humoral and cellular immune responses, inducing antibody production and CD4+/CD8+ T-cell proliferation and activation by either intramuscular or intranasal immunization. MnJ administered intranasally worked as a mucosal adjuvant, inducing high levels of secretory IgA. MnJ showed good adjuvant effects for all tested antigens, including T cell-dependent and T cell-independent antigens [188]. However, despite MnO_2_ nanomaterials having a great potential in the field of biomedicine, a strict biosafety assessment of MnO_2_-based nanosystems is needed in order to comprehensively determine the potential toxicity of Mn-based nanomaterials [189,190,191].

### 4.5. Copper

Regulated cell death (RCD) plays a fundamental role in maintaining physiological homeostasis, including the clearance of aberrant cells. Several studies were devoted to the alternative cancer cell mortality processes associated with inflammation or metal dyshomeostasis, namely necroptosis, pyroptosis, ferroptosis, and cuproptosis; these modalities have been shown to be crucial to cancer therapy effectiveness. In particular, cuproptosis, a new cell death pathway triggered by copper (Cu), was described in 2019 [192] (and the term introduced in 2022 [193]). Cuproptosis is highly associated with the mitochondrial metabolism [194], playing a critical role in tumor cell proliferation, metastasis, and drug resistance [195,196]. In addition, it could be considered as a new strategy in cancer treatment through which to effectively suppress aberrant cell proliferation and cancer metastasis. Notably, MEMO1, an oncogenic protein, was identified as an intracellular Cu-dependent protein, which is required for in vitro breast cancer cell migration and invasion, and in vivo spontaneous lung metastasis [197]. New outcomes have revealed that the administration of elesclomol, or the administration of other copper ionophores, to cancer cell lines affect copper homeostasis and increased Cu(II) levels in mitochondria [198]. This provokes oxidative stress, which leads to ferroptotic death in colorectal cancer cells [199], thus providing evidence of the cross talk between ferroptosis and cuproptosis. Zhang et al. discovered that the key cuproptosis regulator, FDX1, was profoundly downregulated in hepatocellular carcinoma patients, leading to cell resistance to cuproptosis [200].

Given the high levels of Cu in cancer—and the previously reported role of Cu in regulating immune cell function—Voli and co-authors described a role for Cu in regulating the expression of PD-L1 in cancer cells. They demonstrated that copper-dependent anticancer agents, i.e., Cu-chelating drugs, have the potential to be re-purposed for immune checkpoint blockade therapy. More closely, they also showed a strong correlation between the major copper influx transporter copper transporter 1 (CTR-1) and PD-L1 expression across many cancers, the inhibition of the phosphorylation of STAT3 and EGFR, as well as promoting the ubiquitin-mediated degradation of PD-L1. Copper-chelating drugs also significantly increased the number of tumor-infiltrating CD8+ T and natural killer cells, as well as inducing slowed tumor growth and improved mouse survival [201].

It is important to point out that cuproptosis induced by an overload of copper offers great opportunities for exploring the use of copper-based nanomaterials inducing cuproptosis for cancer treatment. The nanoformation of Cu-based nanoparticles (CuO NPs) with intriguing physical, biochemical, and pharmacological properties has been widely studied [202,203]. There is scientific evidence that CuO NPs present dose-dependent toxicity for human breast cancer (MDA-MB 231) and human colon cancer (HCT-116) cells, inducing more apoptosis, ROS generation [204], and alterations in MMPs [202] than those found in normal cells.

Zhang et al., in 2020, showed that, after copper-cysteamine (Cu–Cy)NP intratumoral injections and irradiation via X-rays at the tumor site, groups treated with Cu–Cy+X-ray showed significant inhibitory efficiency from day 12. Cu–Cy NPs-based X-ray-induced oxidative stress in melanoma produces substantial levels of ROS. In addition, it also effectively induces an antitumor immune response, thus enabling simultaneous radio-, oxidative-, and immuno-therapy for the purposes of cancer treatment. The antitumor effect of Cu–Cy-based PDT is facilitated by an increase in the infiltration of immune cells (including DCs, M1 macrophages, CD4+T cells, and NK cells in tumors [205]).

Another example of the use of cuproptosis and copper nanoplatforms in cancer therapy was formulated by Yuzhi Xu et al. These authors showed a 92.4% tumor growth inhibition via an intravenous injection of a glucose oxidase (GOx)-engineered nonporous copper(I) 1,2,4-triazolate ([Cu(tz)]) coordination polymer (CP) nanoplatform. This depletion of glucose (starvation therapy), which was specifically for cancer cells, enhanced cancer cell sensitivity to cuproptosis by reducing the levels of intracellular glucose and GSH, and showed synergistic effects with high specificity and low systemic toxicity [206].

Recent work has shown that copper nanoparticles can be used for nanoparticle-mediated photothermolysis. Thus, photothermolysis, based on near-infrared light-absorbing copper sulfide nanoparticles and 15-ns laser pulses, combined with the immune checkpoint inhibitor anti-PD-1 antibody (αPD-1) increases tumor infiltration via the antigen-presenting cells and CD8-positive T lymphocytes in the B16-OVA mouse model [207]. It was shown that copper nanoparticles synergizes Toll-like receptor 9 agonists and immune checkpoint inhibitors to enhance the abscopal effect in tumors.

Thus, Cu ions and nanomaterials, as well as cuproptosis may have an important role in cancer immunotherapy. Experimental data suggest that cuproptosis, through immune cell regulation, ROS generation, and metal dyshomeostasis, represents a novel antitumor mechanism that paves the way for the possibility of using such NPs to treat tumors, even via the intratumoral administration of Cu-NPs. The possibility of using Cu-NPs as adjuvants is currently limited in its use within a synergy with TLR, and thus requires further research.

### 4.6. Aurum (Gold)

In comparison with other metal NPs, gold nanoparticles (AuNPs) have increasingly attracted the attention of researchers [208,209]. They have been particularly used in biomedical utilization [210,211], from drug delivery [212] to diagnostics and therapeutics. This makes these nanomaterials extremely versatile and promising [213]. It is important to underline that AuNPs can easily be functionalized with antibodies and DNA/RNA in order to specifically target cells [212]; moreover, biocompatible polymers can also be used to prolong their circulation in vivo [108,214]. AuNPs have been explored as immunotherapy carriers in cancer. It has been demonstrated that they are able to improve the delivery and safety of immunotherapy agents; in addition, AuNPs immunotherapies can be used in synergy with photothermal ablation [111].

AuNPs are currently more typical for use in the development of classical subunit vaccines [35,213]. This suggests that such nanoparticles have the properties of adjuvants. AuNPs can work through the EPR effect and can specifically accumulate in tumor tissues and cells, which is highly beneficial for the targeted delivery of tumor vaccines and immune adjuvants [215]. Therefore, AuNPs can be used both as delivery agents and as adjuvants, with the potential of considerably enhancing the immune response with minimum toxicity. Some studies on mucosal vaccines for tetanus toxoid (TT) demonstrated that chitosan-functionalized gold nanoparticles (CsAuNPs), along with plant extracts (Quillaja Saponaria and Asparagus racemosus extracts) as immunostimulants, were able to promote better systemic and local immune responses after oral administration, inducing up to a 28-fold immune response (TT-specific IgG and IgA) when compared to TT and TT-Quillaja Saponaria controls [216,217].

Breast cancer cells express aberrant mucin-type glycans such as α-N-acetyl-D-galactosamine (αGalNAc, the Tn-antigen glycan) [218,219], which are used as a targeting strategy for cancer immunotherapy [220,221]. The Tn-antigen glycan was obtained and converted into well-defined glycopolymers, which were further conjugated to AuNPs, yielding ‘multicopy-multivalent’ nanoscale glycoconjugates [222]. The immunization of a rabbit via these NPs elicited a strong and long-lasting production of antibodies that were selective to the Tn-antigen glycan and cross-reactive toward mucin proteins displaying Tn.

AuNPs functionalized with mucin-1 (MUC-1) glycoprotein, usually overexpressed on epithelial tumor cells, activated peritoneum-derived macrophages that stimulated the release of TNF-ɑ, IL-6, IL-10 and IL-12 [223].

Almeida and colleagues reported that AuNP-OVA injected subcutaneously, without CpG adjuvants, is sufficient to promote significant antigen-specific responses, thus leading to subsequent antitumor activity and a prolonged survival in both prophylactic and therapeutic in vivo tumor models (B16-OVA cells). This finding indicates that AuNPs are effective peptide vaccine carriers with the potential to permit the use of lower and safer adjuvant doses during vaccination [224].

Although AuNPs possess many useful properties, some studies have demonstrated, based on their physicochemical properties, their toxic effects. Sabella et al. showed that the toxicity of AuNPs was related to their cellular internalization pathways [225]. It has also been shown that AuNPs can accumulate in vacuoles, can induce cell death after the treatment of non-cancer cells or after direct injection in the brain [226,227], as well as increase the synthesis of proapoptotic proteins [228]. The safety of AuNPs remains a very controversial issue. In recent studies, researchers have linked the cytotoxicity of AuNPs to their surface charge; the authors also showed a reduction in toxicity by introducing functional groups on the surface of NPs. Moreover, they improved existing synthetic methods and developed new and better methods [229,230,231].

We believe that the possibility of the AuNPs to be used as adjuvants in cancer immunotherapy is the subject of further research. However, special attention should be paid to the safety of their use and the possibility of their accumulation in the body.

### 4.7. Rare Earth Metal NPs

Recently, the nanoparticles of rare earth metals have attracted the interest of scientists. These metals, also known as lanthanides, include elements with atomic numbers ranging from 57 to 71 (e.g., europium, gadolinium, cerium, and yttrium) [232]. Their unique luminescence and magnetic properties make them the metals of choice for the next generation theranostics that efficiently combine the two central pillars of medicine—diagnostics and therapy [233]. A recent review explores the possibility of using such materials for combined immuno- and photo-therapies, including photodynamic therapy (PDT) and photothermal therapy (PTT) [234]. Although PDT could generate certain levels of immune responses by inducing immunogenic cell death and releasing tumor associated antigen (TAA), the immune effects from PDT alone are not enough for inhibiting the remaining tumor growth [235]. Thus, combined PDT and immunotherapy would achieve a superadditive effect, enhancing cancer treatment outcome [236]. Among the lanthanide-based nanoparticles, upconversion nanoparticles (UCNPs) are able to convert near-infrared (NIR) light into ultraviolet and visible light, thus showing excellent photoresponsive performances, which are useful for the phototherapy of deep tumors [237,238]. Some authors have conducted recent studies on synergistic photoimmunotherapy with NIR exposures. In these studies, immunotherapy’s effectiveness was enhanced through the abscopal effect on both tumor metastasis and cancer recurrence [239]. Antigen-loaded upconversion nanoparticles (UCNPs) were used to label and stimulate the DCs, as well as to induce an antigen-specific immune response after being subcutaneously injected into animals [240]. A study by Shao et al. demonstrated that core-shell upconversion nanoparticle@porphyrinic MOFs (UCSs) integrated with α-PD-L1 treatment that were administered subcutaneously promoted the abscopal effect by completely inhibiting the growth of untreated distant tumors via a specific tumor infiltration of cytotoxic T cells [241].

Of particular note is that nanoparticles based on these metals can activate the STING pathway, which is one of the PRRs that are currently being extensively explored [242,243]. In recent years, many STING agonists have been identified and applied to preclinical or clinical trials for the purposes of cancer immunotherapy [244]. The STING pathway is activated by cyclic dinucleotides (CDNs), as is shown in Figure 5. However, CDN-mediated STING activation therapy with LMW compounds is limited by low bioavailability and poor cellular permeability, as well as other adverse effects [245,246]. A promising new study demonstrated that europium nanoparticle formulations of the STING agonist as adjuvants stimulated the type-I interferon (IFN) response in both mouse macrophages and human monocytes, resulting in the maturation of mouse bone-marrow-derived dendritic cells and MHC class I antigen presentation [247]. Subcutaneous administration of europium-based OVA nanovaccines induced a potent humoral response with an increase in primary and secondary anti-OVA antibodies, as well as inducing IL-5, IFN-γ, and IFN-α/β in splenocytes ex vivo. Eu-GAMP-NP–OVA inhibited tumor growth and prolonged survival in B16F10-OVA tumor-bearing mice, promoting cellular immunity with the infiltration of T cells (CD3+) and DCs (CD11c+), as well as inducing a reduction in immunosuppressive cells [247].

Furthermore, new insight into the therapeutic efficacy of lanthanide nanoparticles as adjuvants may come from further research as these nanoparticles seem to have truly promising potential.

## 5. Summary and Concluding Remarks

Breakthroughs in cancer immunotherapy show that the immune system is a good champion against tumor invasion. Despite some successes being achieved and the fundamental rightness of the path being followed, it cannot be denied that there are obstacles for the successful application of this approach in a wider range of patients. Essentially, the main concerns are associated with increased toxicity when multiple immunomodulators are administered. Based on the previous conclusion regarding the simple solutions [248] that are to be adopted for cancer therapy, we propose that the local administration of a drug combining an immunomodulatory agent and an adjuvant could be a profound solution. MNPs seem to be the best candidates for this purpose. This is facilitated by their inherent properties, such as local immunogenicity and retention in tissues, as well as the possibility of introducing new specified properties through structural modifications. Given that innate immunity is the key to the induction of robust adaptive immunity, and that the immunosuppressive TME is the main hindrance to overcome, intratumoral immunotherapy—which is where the delivery of immunostimulants into the tumor is combined with an in situ activation of innate immunity—is a rational strategy [249].

Until recently, cancer therapy based on the intratumoral administration of drugs was considered very unusual for practicing oncologists. Now hundreds of clinical trials are devoted to it. Drugs administered intratumorally can be divided into three large groups: viral, cell therapy, and immune system activators (which mainly act as TLR agonists). Toll-like receptors (TLRs) play crucial roles in the innate immune system by recognizing pathogen-associated molecular patterns. Thus, TLRs are excellent targets for adjuvants to provide a “danger” signal to induce an effective immune response that leads to long-lasting protection. MNPs are exogenous molecules that have the property of activating the “danger” signal and can act as adjuvants for cancer immunotherapy. Despite the fact that a fairly large number of MNPs are in various studies, their use in cancer therapy CTs is rather limited. Historically, they have been more commonly used as delivery molecules or, more recently, as agents for the precise imaging of tumor foci.

We analyzed the properties of some MNPs and noted works that directly indicate the possibility of their use as adjuvants, including in the context of cancer therapy (summarized in Table S2). A few studies in animal models have shown that the combination of MNPs with various immune-system-activating proteins (cytokines) can provide a greater therapeutic effect than these components alone. Moreover, in 2022, the authors noted the possibility of such a complex system affecting distant metastases [136]. We suggest that such studies will only progress in the future since they offer a reasonably safe alternative to systemically administered drugs. Since the goal of intratumoral immunotherapy is to prime immune cells locally in order to generate a systemic antitumor effect, then using the tumor as its own vaccine will allow one to generate an antitumor immunity against multiple cancer cell antigens without having to pre-identify those antigens [250]. Thus, intratumoral immunotherapy can potentially elicit a polyclonal antitumor immune response against multiple concurrent targets and could provide a broad attack against tumors and their metastases.

The combination of an immunotherapeutic agent and MNPs as an adjuvant may be promising from the point of view that the combination of multidirectional therapies can produce a synergic or enhanced effect. Such speculation comes from the reinforcement effect when two toxic compounds are used. If the effect of the action of two different drugs Tx(a) and Tx(b) is greater than their arithmetic sum ΣTx(a,b) > Tx(a) + Tx(b), then such an effect will be a “superadditive effect”. The concept of additivity was established a long time ago and further developed when it was shown that the combination of radiotherapy and chemotherapy provide improved cancer treatment outcomes due to superadditive effects [251,252]. In general, the best result is achieved when both toxic agents enter the target cancer cells simultaneously [253,254]. It is assumed that the combination of two agents in one hybrid bifunctional nanocomplex allows it to synergistically increase its therapeutic efficacy due to the superadditive effect [255]. For gene therapy, we have shown a similar effect when using a suicide gene and an immunoactivator gene in one genetic construct [256].

Extensive research on MNPs is driven by the enormous possibilities of their modifications and applications. Their ability to induce an immune response is becoming increasingly prevalent in cancer therapy. We have considered, as a particular example of such an immune response, the ability of MNPs to act as adjuvants, specifically in terms of inducing a non-specific activation of the immune system. Such an effect has previously been widely considered for the development of infectious disease vaccines [156]. We believe that this experience can be successfully translated into antitumor therapy. This is supported by the possibility of achieving a superadditive effect when using MNPs as an adjuvant, as well as the additional use of an immune activator. At the same time, if immune checkpoints are used, then the adjuvants can be used to overcome a low response of such therapy and/or to reduce systemic toxicity. In combination with other immune activators, it is possible to anchor them in the tumor due to the properties of MNPs, which also reduce systemic toxicity and increase the local concentration of the drugs. For fast and successful studies aimed at finding combined drugs, we suggest that the following prerequisites be followed:

− Local administration;
− Using MNPs that are known and well studied;
− Paying special attention to safety/toxicological studies;
− If possible, using MNPs to anchor immunoactivators in tumors in order to avoid entering the bloodstream and causing systemic toxicity;
− An abscopal effect must necessarily be achieved when the drug is administered locally;
− Using scalable and reproducible MNP production.

The following concluding remarks are based on both the versatile nature of NPs and the specifics of drug use:

NPs for clinical use must meet FDA or other regulatory standards. Before introducing NPs for cancer treatment, a complete evaluation of their pharmacodynamics and biodistribution profiles are necessary in order to avoid possible toxicity. Biodistribution affects the off-label on-target toxicity of drugs, and differences in pharmacodynamics affect the possibility of an abscopal effect.

The last crucial aspect to consider in conducting a possible cancer immunotherapy based on the combination of immunomodulators and MNPs as an adjuvant should be the evaluation of their tolerability at repeated doses. In other words, how many times can one give the drug to a patient before the immune system will neutralize it.

Thus, we suggest that the development of hybrid complexes and compounds based on MNPs and immunomodulators is at an early stage of development. The use of MNPs as adjuvants should solve the problem of the toxicity of multiple immunomodulators. At the same time, the properties of the metal as an adjuvant are designed to solve the problem regarding the presence of an abscopal effect for locally administered cytokines. If a combination of “non-abscopal cytokine” with MNPs is able to provide abscopal effects, then it would benefit cancer immunotherapy.

## Data Availability

The data presented here is available throughout the article and in the Supplementary Table S1.

## Supplementary Materials

The following supporting information can be downloaded at: https://www.mdpi.com/article/10.3390/pharmaceutics15051346/s1, Table S1: Clinical Trials; Table S2: Evidence level for MNPs and metal-based substances to be used as adjuvants.
