# Supplementary material for: Prospects for the Use of Metal-Based Nanoparticles as Adjuvants for Local Cancer Immunotherapy
Source: Pharmaceutics. 2023 Apr 27;15(5):1346. doi: 10.3390/pharmaceutics15051346 (PMC10222518; doi:10.3390/pharmaceutics15051346)
Supplement: Supplementary file 1 [file pharmaceutics-15-01346-s001.zip › Supplementary Table S2_Revised.pdf]

Table S2. Evidence level for MNPs and metal-based substances to be used as adjuvants

Metal based NPs		Model and administration route	Evaluation of results	Ref
**Alum**	Al-NPs	Mouse s/c injection at the root of tail or right flank or i/m injection in the calf muscles of hind limb	Adjuvant for developing vaccines for cancer immunotherapy	[128-130]
	Al-NPs (nanosized AlO(OH)	RAW264.7, DC2.4, BMDC cells	Deliver antigen and adjuvant to APC in lymph nodes; augment antigen-specific CD8+ T cell responses and promote Th1 type immunity	[131, 132]
	Alhydrogel® and stabilized rod-shaped Al(OH)3-NPs (~60 nm) from Alhydrogel®	Mouse i/m injection in the calf muscles of hind limb	Elicited a robust TH1 immune response characterized by antigen-specific CD4+ T cells expressing IFN-γ and TNF, as well as high IgG2 titers	[130]
	Aluminum adjuvant-integrated nano-Metal- Organic Frameworks (MOF)	Mouse s/c injection	Enhance humoral and cytotoxic T lymphocyte immune responses, accumulate in lymph nodes, uptake by DCs	[134]
	Alum-tethered cytokines	Mouse intracranial injection	Fusion to cytokines enhance immune response and survival	[135]
	Aluminum hydroxide (alum) and recombinant cytokines NPs	Mouse i/t administration	Alum-bound IL-12–ABP vs IL-12– ABP – increase tumor retention, response to therapy and survival, safety	[136]
	Alum-ABP-IFNα	Mouse i/t administration	Similarly to [137] improves treatment efficacy and reduces toxicity	[138].
**Ferrum**	IONPs	Mouse	Complement system activation, immune cell recruitment, and inflammasome activation	[154]

	IONPs	Mouse intratracheal instillations of 50uL each	Adjuvant outcome on Th2 response	[155]
	Mycobacterium tuberculosis vaccine with IONPs	Mouse s/c injection	Adjuvant enhanced the response of specific Th1 (CD4+ IFN- γ), Th17 (CD4+IL-17+) and TCD8+ cells	[156]
	IO-RNA-NPs	DC2.4 murine dendritic cells, B16F10-OVA murine melanoma cell; mouse	Ex-vivo DC activation; enhanced activation of antigen-experienced T-cells and priming of naïve T- cells in an antigen-specific manner	[157]
	Fe3O4 NPs	Mouse s/c injection	Enhance the H22 autovaccine	[158]
	Fe3O4-OVA NPs	Mouse i/t administration	Humoral and cellular immune responses in BALB/c mice	[159]
**Titanium**	TiNPs with nickel (nitinol)	Mouse i/p injection	Induce rapid expansion of T cells in (CAR)-T therapy	[172]
	Spike TiO2	Mouse breast cancer 4T1 cells; mouse i/v administration	Exhibited immunomodulatory effects and stimulated TNF- and IL-2 in a time- and concentration- dependent manner	[177]
	HTiO2 NPs	Mouse i/v administration	Demonstrate immunomodulatory effect with high levels of IL-6, IL- 1β, TNF-α	[178]
**Manganese**	tadpole-ovoid manganese- doped hollow mesoporous silica coated gold nanoparticles (Au@HMnMSNs)	Murine breast cancer (4T1) cells; mouse i/v injection	Enhance the induction of immunogenic cell death and maturation of dendritic cells; enhance the intratumoral infiltration of cytotoxic T lymphocytes (CTLs)	[183]

	(DOX)-loaded and phospholipid (PL)-coated hybrid NPs (PL/APMP-DOX NPs)	Mouse breast cancer 4T1 cells; mouse s/c injection	Boosts dendritic cell maturation and increased cytotoxic T lymphocyte infiltration into the tumor site	[103]
	Mn-phenolic network NPs	macrophages; mouse	Internalize into macrophages and stimulate M1 polarization; successfully inhibited tumor growth and prolonged survival	[187]
	CDN-Mn2+ Particle, CMP	CT26 tumour-bearing BALB/c i/t administration	CDN STING agonist	[66]
	MnJ, Mn Jelly	B16-OVA mouse model i/t immunisation	STING agonist	[188]
**Copper**	CuS	B16-OVA mouse model	synergizes Toll-like receptor 9 agonists and immune checkpoint inhibitors	[207]
**Aurum**	AuNPs	Different models	classical vaccines	[35, 213]
	Tn-antigen glycan-AuNPs	Rabbit s/c injection	Long-lasting production of antibodies	[222]
	AuNPs functionalized with MUC-1 protein fragment	Primary peritoneum-derived macrophages	Induce macrophage activation, increase antigen presentation and predominant M1 polarization	[223]
	AuNP-OVA without CpG adjuvants	B16-OVA cells; mouse s/c injection	antigen-specific responses	[224]
**Rare earth metal NPs**	Antigen-loaded UCNPs	Mouse s/c injection	Stimulate dendritic cell , induce an antigen-specific immune response	[236]

	Core-shell upconversion nanoparticle@porphyrinic MOFs (UCSs)	Mouse s/c injection	Inhibit the growth of untreated distant tumors via specific tumor infiltration of cytotoxic T cells	[237]
	Europium-based OVA nanovaccines	Mouse s/c injection	STING agonist	[247]

Abbreviations: s/c, subcutaneous; i/m, intramuscular; i/t, intratumoral; i/p, intraperitoneal; i/v, intravenous.
